
JOURNAL INFORMATION
==============================
NLM Title Abbreviation: J Orthop Traumatol
Journal ID: J Orthop Traumatol
NLM Journal ID: 101090931

Journal of Orthopaedics and Traumatology : Official Journal of the Italian Society of Orthopaedics and Traumatology

ISSN: 1590-9921
EISSN: 1590-9999
Publisher: Springer

ARTICLE INFORMATION
==============================
PMCID: PMC4417792
DOI: 10.1007/s10195-013-0257-8
Article ID: 257
Article version: 1
Subjects: Events

Oral Communications

Electronic publication date: 2013 Sep 18
Print publication date: 2013 Oct
Volume: 14
Issue: Suppl 1
First page: 1
Last page: 11
Copyright: © The Author(s) 2013
License: Open AccessThis article is distributed under the terms of the Creative Commons Attribution 2.0 International License (https://creativecommons.org/licenses/by/2.0), which permits unrestricted use, distribution, and reproduction in any medium, provided the original work is properly cited.
License URL: https://creativecommons.org/licenses/by/2.0/

Keywords: Total Knee Replacement, Acetabular Fracture, Proximal Humeral Fracture, Tibial Plateau Fracture, Proximal Humerus Fracture
issue-copyright-statement: © The Author(s) 2013

==============================
J Orthopaed Traumatol (2013) 14 (Suppl 1):S1–S11
DOI 10.1007/s10195-013-0257-8

26 OCTOBER 2013

### Oral Communications

### ORAL COMMUNICATIONS

### *C01–TRAUMATOLOGY 1*

### Skeletal injuries in the emergency: mistakes and overlooking

G. Baumgartner*[1], S. Pelle[2], D. Longo[2], M. Almberger[3], P. Braidotti[4], E. Sacco[1]

[1]UOD Ortopedia d'Urgenza, Policlinico Umberto I (Rome, IT); [2]Dipartimento Scienze Anatomiche Istologiche Medico-Legali e dell'Apparato Locomotore, Università degli Studi di Roma "La Sapienza" (Rome, IT); [3]UOC Radiologia Generale e d'Urgenza, Policlinico Umberto I (Rome, IT); [4]UOD Ortopedia d'Urgenza, Policlinico Umberto I (Rome, IT)

**Introduction** Decision-making in ER Department has unique features compared with normal diagnostic procedures in a typical setting like routinely examination of both inpatients and outpatients. Limited time for history, examination and imaging in addition to environmental factors like busy hours, tiredness, fatigue, emotional response facing serious injuries all contribute to missed diagnosis and litigation.
**Materials and methods** In this paper, we scrutiny a definite set of skeletal injuries occurred in our ERD since 2002 that are historically established as most challenging diagnostic traps. Analyzing the cases, we were able to point out to precise factors of error in order to create a mental map to avoid them.
**Results** Overlooking basically rests on three mechanisms: (1) subtle, subliminal lesions (e.g. hairlike fracture line in toddler's fracture); (2) attention-seeking lesions in association with 'modest' ones (e.g. diaphyseal femoral fractures + ipsilateral hip dislocation; proximal ulnar fracture + ipsilateral radial head dislocation in paediatric elbow; shoulder dislocation + ipsilateral coracoid fracture; medial malleolar fractures in Maisonneuve's fractures); (3) rare lesions often associated with inadequacy of the standard roentgenological views (e.g. posterior shoulder dislocation; humeral distal epiphyseal separation in the newborn).
**Discussion** We stress in addition that these lesions do not always eventually turn, if overlooked, into permanent damage but someone do yield severe complications, especially in children. Besides, a prompt and early diagnosis makes the treatment easier while the late treatment is often plagued by overwhelming difficulties.
**Conclusions** In conclusion, a high grade of suspicion is necessary whenever the above mentioned circumstances come up. As Kant says, however, the seed of suspicion growths in the ground of knowledge; therefore, we can recognize them only is we are capable to learn the experiences of our masters and keep sharing this information within the scientific community.

### The dynamization of locked nails: when, where and how it should be performed

G.B. Morace*, A. Carraro, P. Esopi

A.S.L. 13 Dolo (Dolo, IT)

**Introduction** The dynamization of locked nails is still a debated and not completely clarified question. It is a simple surgical gesture whose actual usefulness is often questioned.
**Materials and methods** Our object is therefore to throw light on the topic, proposing some guidelines for a correct application of this procedure and answering the following questions: WHEN it should be done—that is, in which fractures and phase of treatment; WHERE it should be done—whether on the proximal or distal extremity of the locked nail; and finally, HOW it should be done—whether totally removing the locking screws from a nail extremity, or maintaining the screw in the dynamic hole.
**Results** An accurate analysis has taken into consideration the degree of stability and the coaptation of the fracture stumps, the possible migration of the locked nail, the surgery mistakes, and other possible factors that may condition the reparative process. In most cases, where the nailing operation has been accurately performed, the dynamization of a locked nail turns out to be unnecessary.
**Discussion** However, the dynamization may be useful to compensate for possible surgical mistakes, and in case of delayed consolidation. Two fundamental criteria must be considered for a possible dynamization: the degree of fracture stability, and the possible sliding of the locked nail.
**Conclusions** Finally, in some cases, pre- and intrasurgical planning must consider the opportunity of a following controlled dynamization.

### Definitive treatment of articular fractures with external fixing

G. Pristerà*, N. Ruggieri, C. Palumbo

SCDO Ortopedia e Traumatologia, Presidio Molinette, Città della Salute e della Scienza di Torino (Turin, IT)

**Introduction** The right articular surface reconstruction in an articular fracture is the aim to avoid an early post-traumatic osteoarthritis. In tibial plate fractures is often difficult to obtain a good articular surface reduction because the bundling of the tibial metaphyseal cancellous bone trabeculae produce a lowering of the tibial plate and a bone loss under it. For this reason, in these fractures we try to obtain the articular surface as anatomically as possible and reconstructing the underlying bone by osteosynthesis and by using bone substitutes. We wanted to assess how much articular reconstruction was able to avoid a symptomatic osteoarthritis.
**Materials and methods** Between January 2000 and December 2008, 63 closed tibial plate fractures underwent to surgical treatment. They were 28 females and 35 males with an average age of 50 years old (20–80). According AO classification, there were 38 41B fractures and 25 41C. The reconstruction of the articular surface, obtained pushing upward the tibial plate, was monitored with fluoroscopy and arthroscopy. The osteosynthesis was performed in the majority with plate and screws (57) while only in 6 cases it was used only free screws. In almost all the cases (59), the reconstruction was completed by bone substitutes (Norian, Callos) in areas of metaphyseal bone loss for the best support of the tibial plate. At an average follow-up of 138 months, these patients were clinically and radiographically checked to highlight the onset of knee osteoarthritis.
**Results** Radiographic evaluation of osteoarthritis, present in 57 cases, was estimated according to Alback, with 38 cases with radiographic I osteoarthritis, 15 of II and 6 of III. The clinical evaluation, according to Knee Society Score, checked 4 excellent cases, 21 good cases, 17 discrete cases and 21 poor cases.
**Discussion** The tibial plate fractures, despite a thorough joint reconstruction, at a later time, in one-third of cases, presents significant symptomatic and radiological joint degenerations. The joint degeneration occurred even after an adequate articular reconstruction in connection to the type of fracture, meniscal lesions and age of the patient.
**Conclusions** The tibial plate fractures require an accurate anatomical reconstruction and a good osteosynthesis. Despite this, the major articular alterations in older age lead to a crippling arthritic changes which often will require a joint replacement.

### Early experience of non-contact bridging (NCB) periprosthetic plate in complex periprosthetic fractures of total hip replacement (THR) and total knee replacement (TKR)

A.S. Ghatahora[1], N. Daud[1], A.B. Ng[1], D. Pili*[2]

[1]Pinderfields General Hospital (Wakefield, UK);
[2]Peterborough and Stamford Hospitals (Peterborough, UK)

**Introduction** The treatment of periprosthetic fractures is always problematic and, when fixation is the indicated treatment, choosing the right implant is as well very difficult. We present our preliminary experience of the treatment of complex periprosthetic fractures of the femur in order to evaluate the clinical outcome and early experience of NCB (2nd generation) polyaxial locking plates in periprosthetic fractures of THR and TKR without cortical strut graft.
**Materials and methods** Between January 2011 and July 2012, 11 cases of complex periprosthetic fractures of THR and TKR were prospectively reviewed. There were 2 males and 9 females with mean age of 80.7 years old (range 70–88) with an average follow-up of 10.2 months (range 7–16). The Vancouver and Rorabeck systems were used to classify fractures around THR and TKR respectively. All patients had clinical, radiographic assessment and underwent a patient satisfaction survey at 6 weeks, 3 months, 6 months and 1 year postoperatively.
**Results** There were 11 cases of periprosthetic fracture of THR; Vancouver C (n = 5), Vancouver B2 (n = 2), Vancouver B1 (n = 3) and 1 case of periprosthetic fracture of TKR; Rorabeck II (n = 1). There were three cases of interprosthetic fracture of the femur. All patients demonstrated clinical and radiological union of fractures at latest follow-up and all made a full return to pre-injury activities of daily living (ADLs). There were no cases of infection, mal or nonunion. No other complications were noted. All patients were extremely satisfied with their clinical outcome.
**Discussion** The incidence of periprosthetic fractures is rising due to the increasing numbers of implants, the lengthening of the average life expectancy and the increased functional expectations of our patients. The typical patient is usually elderly, with several comorbidities and often under steroids. Technically, the treatment is often extremely challenging due to the fracture configuration and bone quality. Choosing the right implant is often difficult. A good implant, in order to be employed in the most possible fracture configurations, should be easy to use and very versatile.
**Conclusions** The early experience and clinical outcome of this prospective study suggest that NCB (2nd generation polyaxial locking plate) periprosthetic plate is a viable option for complex periprosthetic fractures around THR and TKR without the use of cortical strut allograft. The short-term outcome is particularly promising in complex interprosthetic fractures of THR and TKR.

### *C02–TRAUMATOLOGY 2*

### The articular reconstruction in complex acetabulum fractures: functional and clinical results in our personal experience

A. Musetti, M. Pagliari*, S. Del Sordo, E. Demattè

Ospedale S. Chiara (Trento, IT)

**Introduction** In our study we analyzed prospectively the results of patients with complex dislocated fractures of the acetabulum treated over a period of 4 years (from 1 January 2008 to 1 January 2012).
**Materials and methods** We included 55 patients, 44 were males and 9 were females, aged 28–83 years (mean 38 years). In 16 cases a central dislocation of the femoral head was associated. The fractures were classified using the classification of Judet–Letournel. During operation, a Kocher Langenbeck approach was used in 28 cases, an anterior ilioinguinal approach in 16 cases, a combined anterior and posterior approach in 7 cases. An external fixation was used in two cases (as a wait treatment in two elderly patients with osteoporotic bone). Mehne and Matta plates were used in all cases. AP and Judet oblique radiographs and a CT scan often with 3D reconstructions were taken preoperatively and during follow-up. Clinical results were evaluated according to the Modified Grading System of Merle d'Aubigné, while radiological assessment were analyzed according the Matta X-ray evaluations criteria.
**Results** The mean follow-up was 36 months (12–58 months). Functional results according to the modified scale of Merle d'Aubigné were excellent in 15 cases, good in 29 cases, fair in 9 and poor in 2 cases. According to Matta radiological evaluation, anatomic reduction was excellent in 14 cases, good in 26, fair in 13, poor in 2 cases. There were seven cases of post-traumatic arthrosis of which four were subjected to subsequent THR; two cases of deep venous thrombosis and seven cases of avascular necrosis of the femoral head.
**Discussion** The affecting factors of functional results of complex acetabulum fractures are many: fracture types, associated injuries of the femoral head, timing of surgery, an older age of the patients especially with the presence of osteoporosis, associated ipsilateral lower limb fractures, the presence of dislocation of the femoral head. However in patients who have a complex acetabulum fracture the hip joint can be preserved and post-traumatic osteoarthrosis can be avoided only if an anatomical reduction is achieved and in fact the clinical result is related closely to the radiographic result.
**Conclusions** An increase in the rate of anatomical reductions in this type of fracture and the correct timing of the surgical procedure should be the goals of surgeons who treat these complex fractures.

### Acetabular fractures in elderly patients: our experience

G. Longo*, F. Carluzzo, S. Bonfiglio, G.C. Salvo, S. Milazzo

A.O. Cannizzaro (Catania, IT)

**Introduction** The acetabular fractures in the elderly are characterized by a high variability in terms of types of patients and fracture characteristics. In the elderly, the majority of acetabular fractures are pathological fractures due to the presence of osteoporosis, caused by falling low energy and can occur in unusual way.
**Materials and methods** At A.O. Cannizzaro of Catania from 2007 to 2012, we treated 45 patients with acetabular fractures, over the age of 60 years. Of these patients, 34 were treated surgically (31 patients with ORIF, 3 patients with ORIF more acute PTA) and 11 conservatively. Of the patients treated surgically only 21 were subject to follow-up clinical, radiographic and psycho-functional (SF-12 card), while 9 patients were evaluated only with card SF-12. Of the 31 patients surgically treated with ORIF only 4 needed a second operation of PTA secondary to the development of hip osteoarthritis post-traumatica.
**Results** The range of movement of patients surgically treated with ORIF was similar if compared with contralateral hip or with patients treated conservatively, we witnessed only a modest reduction of intrarotation respect to the contralateral hip. The results of the functional and psychological SF-12 as well as the quality of life were better in patients surgically treated with ORIF compared to those subjected to synthesis more PTA in acute or secondary hip prosthesis for the development of a post-traumatic arthrosis.
**Discussion** The anatomical reconstruction of the acetabulum, as demonstrated in the literature, it is clearly preferable to the prosthetic replacement since it allows to avoid many of the well-known complications due to surgery of the hip prosthesis, although the anatomical reconstruction of the acetabulum can considerably more complex than in the elderly patient for the fracture characteristics that distinguish it. A non-surgical treatment can be justified only if there is no serious breakdown of the acetabulum, or hip subluxation in the absence of traction and in patients with poor general condition.
**Conclusions** In the literature, the treatment of acetabular fractures in elderly patients is very controversial (ORIF vs. arthroplasty in acute vs conservative treatment). In our experience the ORIF represents an excellent treatment option in acetabular fractures in elderly patients and, in patients who do not develop secondary osteoarthritis were documented excellent results both from the clinical point of view that quality of life.

### *C03–TRAUMATOLOGY 3*

### Lesser trochanteric avulsion in trochanteric fracture: do we need to fix it in young patients?

A. Aprato*[1], R. Lo Baido[2], A. Crosio[2], R. Matteotti[2], E. Grosso[3], A. Massè[1]

[1]Orthopaedic Department, School of Medicine, San Luigi Hospital, University of Turin, Orbassano (Turin, IT);
[2]Orthopaedic Department, School of Medicine, University of Turin (Turin, IT);
[3]Department of Traumatology, C.T.O. Hospital of Turin (Turin, IT)

**Introduction** Lesser trochanter avulsions are often associated with a pertrochanteric or intertrochanteric fractures. Aim of this study is to evaluate the possible strength loss after this kind of avulsion in pertrochanteric or intertrochanteric fractures after closed reduction and intramedullary fixation.
**Materials and methods** Patients with a consolidated intertrochanteric fracture associated or not with lesser trochanter avulsion were enrolled respectively in group A and group B. Criteria of inclusion was the achievement of an anatomic reduction with gamma nail and a complete consolidation of the fracture. Criteria of exclusion were: a follow-up shorter than 1 year and age over 65 years old at surgery. Patients were retrospectively review for the purpose of this study. Range of motion, modified Harris hip score (HHS), flexion strength with hip in neutral position and at 90° of flexion were evaluated on injured and healthy side. On the X-rays the displacement of the lesser trochanter was calculated.
**Results** Group A and B showed no statistical difference in age and follow-up. No statistical difference between the two groups was found in range of motion, mean mHHS, hip flexion strength at 90° of hip flexion. Lesser trochanter avulsion group showed a significantly reduced strength in flexion with hip in neutral flexion (mean difference between two groups was 18.5 N). Lesser trochanter displacement showed a significant correlation with strength at 90° of flexion.
**Discussion** Only one study has been published regarding this topic but it presents several limits and lacks of statistical analysis.
**Conclusions** Our results suggest that, although an avulsion of the lesser trochanter in a trochanteric fracture leads to a significant loss of strength, the strength reduction is limited and fixation of the lesser trochanter should be performed only in high demanding patients.

### Risk of mortality related to proximal femur fractures in elderly

M.F. Surace*[1], S. Fozzato[1], L. Monestier[1], J.M.T.A. Meessen[2], S. Pisani[3], M.L. Gambino[3], D. Bonarrigo[3], P. Cherubino[1]

[1]Clinica Ortopedica, Dipartimento di Biotecnologie e Scienze della Vita, Università dell'Insubria (Varese, IT);
[2]Vrije Universiteit (Amsterdam, NL);
[3]Osservatorio Epidemiologico ASL (Varese, IT)

**Introduction** Fractures of proximal femur are frequent in the elderly. Literature reports 12–36 % risk of mortality within first post-operative month. The objective of this study is to highlight correlations between many variables and risk of mortality, in patients registered on Varese provincial and hospital database.
**Materials and methods** 69–99 years old patients with primary (not pathological) proximal femur fracture were included. Assessment was based on demographic (age, sex, marriage), anamnestic (co-morbidities, Charlson index score, smoking, alcohol abuse, ASA score, BMI) and treatment aspects (hospital, treatment choice, surgery and duration of hospital stay, anesthesia). Correlation between variables and risk of mortality was examined by the means of Kaplan–Meier Survival Graphs and Cox Proportional Hazards Analysis.
**Results** Eight hundred and twenty-eight cases of Varese provincial database were included: risk of mortality increased in 84–99 years old male patients with Charlson index score >1. No significant correlations were documented with other examined aspects. One hundred and ninety persons in Ospedale di Circolo di Varese database were included: risk of mortality increased in 84–99 years old male patients who underwent surgery after 48 h. Hypertension and diabetes increased the risk of mortality, while cardiovascular co-morbidities reduced it.
**Discussion** Some factors are highlighted in both databases: age, sex, co-morbidities considerably influence risk of mortality in patients with proximal femur fractures. Results about influence of cardiovascular co-morbidities are controversial: it could reasonably stated that this group of patients undergo continuous monitoring of the health status even before surgery, reducing peri-operative complication rate.
**Conclusions** Risk of mortality due to proximal femur fracture increase in old patients with co-morbidities; early treatment is essential in order to reduce complications.

### A new divergent locked screws system for treatment of pertrochanteric fractures

P. Palombi*[1], A. Palombi[2]

[1]Casa di Cura Villa Mafalda (Rome, IT);
[2]Dottorato di Ricerca presso Università "Tor Vergata" (Rome, IT)

**Introduction** It is introduced the divergent locked screws system for the treatment of pertrochanteric fractures (AO/OTA 3.1.A1 and A2): it is characterized by the biomechanical advantage of shortening the lever arm between the gravitary axis bearing on the femoral head and the fulcrum of the osteosynthesis system, allowing a robustness osteosynthesis reached by mini-invasive approach. Surgical technique is shown. Aim of this paper is to evaluate the new system and compare its preliminary results with the current literature.
**Materials and methods** From July 2009 to February 2012, 240 proximal femoral fracture (AO/OTA 31.A1 and 31.A2) were treated with divergent locked screws system. One hundred seventy-six of them (113 women and 63 men) have been included in the follow-up term of 2, 5, 13 weeks, and then at 6, 12 and 18 months.
**Results** All patients had good or excellent midterm results evaluated by objective and subjective scores. Objective scores were assessed by orthopaedic surgeons on the basis of clinical and radiographical assessment, intra- and post-operative blood loss, operative time, intraoperative X-ray exposition, rate of complications and failure, time to the full weight bearing. Subjective scores were assessed by surgeons and patients on the basis of restored function, restored quality of live and overall satisfaction.
**Discussion** The divergent locked screws system showed to be a powerful and cost-effective alternative in the treatment of stable (AO/OTA 31.A1) and unstable (AO/OTA 31.A2) pertrochanteric femoral fractures. It showed easy intraoperative management of fracture fragments, very good stability, and rapid bone healing.
**Conclusions** Extra-articular proximal femoral fractures are very common and related to high to life and to life quality, as well as social and economic costs. With population aging they are going to be more and more frequent, and reducing their risks and costs is a real priority for orthopedic surgery. Many type of osteosynthesis are known to be safe and effective; the new system seems to be a good innovation able in improving results.

### *C04–TRAUMATOLOGY 4*

### Management of the proximal femoral metaepiphysis fractures in patients over 65: the importance of early treatment and follow-up

V. Sessa[1], S. Barreca[2], S. Costa[1], L. Micciché[2], P. Beatrice*[1], M.A. Rosa[2]

[1]Fatebenefratelli—Isola Tiberina (Rome, IT);
[2]Scuola di Specializzazione in Ortopedia e Traumatologia, Sezione di Ortopedia e Traumatologia, Dipartimento di Scienze Biomediche e delle Immagini morfologiche e funzionali, Università degli Studi di Messina (Messina, IT)

**Introduction** Fractures of the femoral neck are a widespread disorder because of increasing people average age. Treatment is hard, because of bad bone quality and poor general conditions. The goal of this study is to evaluate the relationship between the waiting time for surgery and the outcome/death rate of treated patients.
**Materials and methods** We studied hip fractures between 2007 and 2011; we considered age (>65 years), sex, fracture type, treatment type, time between hospitalization and surgery, death rate around surgery. Lateral fractures (58 %) has been treated with intramedullary nail; Medial fractures (42 %) has been treated as follow: Garden type 1 and 2 (23 %) with Asnis screws, Garden type 3 and 4 with total hip arthroplasty (7 %) in good general condition patients and hip bipolar hemiarthroplasty (70 %). We valued results through X-ray and clinical parameters HHS and we considered if patients followed an anti-osteoporosis therapy.
**Results** The follow-up is included between 18 months and 5 years. We treated 394 hip fractures (76 % female and 24 % male). Average age is 80 years and 9 months. Average time between hospitalization and surgery is 49 h. Death rate within 1 year from fracture is 17.5 %. Average HHS is 74.5:76.2 in patients treated within 48 h and 72.7 in patients treated after 48 h. All patients got a home anti-osteoporosis therapy but 57 % of patients suspended it without medical authorization.
**Discussion** The annual incidence of hip fractures is increasing because of aging of population. The goal is to reduce waiting time for surgery to get the best results and to reduce the social and economic impact of the pathology. We need to perform a fine preoperative evaluation to get patient better recovery.
**Conclusions** In our series data about age, sex, fracture type and treatment type are not different from national and international data, we only got a lower death rate (17.5 % within 1 year) than the average value of other international studies (25–30 %).

### A new locking plate and dynamic screw system for internal fixation of intracapsular hip fractures; results for the first 530 patients treated

M. Parker, D. Pili*

Peterborough and Stamford Hospitals (Peterborough, UK)

**Introduction** The Targon Femoral Neck Hip Screw has been designed to improve the fixation of intracapsular hip fractures. Fracture healing complications after internal fixation occur in approximately 10 % of undisplaced fractures and 30–50 % of displaced fractures. The new implant consists of a small plate with six locking screw ports. The two distal holes are used to fix the plate to the lateral cortex of the femur. Three of four screws are passes through the proximal holes and across the fracture site. These 6.5 mm screws are dynamic to allow for collapse of the fracture across the femoral neck. A jig is used to aid insertion of the device with minimal surgical exposure of the femur.
**Materials and methods** For the first 518 patients treated with this implant at the first centre to use this implant, the mean age of the patients was 76 years (range 22–103), 58 % were female. The mean length of surgery was 46 min and the mean length of anaesthesia 59 min. The median length of institutional stay till discharge home was 9 days (mean 13 days, range 1–107). Four telescoping screws were used in 55 % of patients, 3 in 44 % and 2 in 1 % of patients.
**Results** Follow-up of patients at present is a minimum of 6 months. For the 185 undisplaced fractures there have been 6 cases of non-union and 6 cases of avascular necrosis. For the 233 displaced fractures (Garden III and IV) there have been 42 cases of fracture non-union, 25 cases of avascular necrosis and 5 cases of plate detachment from the femur treated by repeat fixation. In addition there was one deep wound sepsis treated by removal of the implant and girdlestone arthroplasty.
**Discussion** Observation of those fractures that have healed shows there has been between 0 and 22 mm of collapse at the fracture site which occurs along the line of the femoral neck. There has been no tilting of the fracture into varus as occurs with a parallel screw method.

**Conclusions** The results to date show an incidence of fracture healing complications is about a half that which is to be expected with a parallel screw method. This new implant may be a significant advance in the treatment of this difficult and common fracture.

### Advantages of the Affixus nail in 31A fractures of the proximal femur

P. Sanna*, S. Ghera, I. Cerocchi, M.G. Minicelli

Ospedale San Pietro (Rome, IT)

**Introduction** Femoral neck fractures are increasing in number. Nailing is often the preferred choice in the treatment of this type of fractures, with very good mechanical performance and early functional recovery of the patient. The technical innovations introduced with the Affixus nail are here presented. Surgical instrumentation is easier and more simple, with a linear step by step procedure, allowing fast and safe nailing.

**Materials and methods** From June 2011 to January 2013, 76 nails were employed, in our department, in 31A1, 31A2 and 31A3 fractures (according to AO classification). A short (180 mm) and long (from 260 to 460 mm) nail are available, with 125° and 130° angulation. A proximal lateral bending of 4° and a 10° anteversion are present. The diameter is 15.6 mm proximally and 9, 11 and 13 mm distally.

**Results** Fracture healing was obtained in all patients, with a delayed union in 8 cases (6 31A3 and 2 31A2). No nail breakage was observed. Antirotational screw was used only in 31A1 cases in which a very proximal rim was seen. In 31A1 fractures a standard nail was used without distal locking screws. In very dislocated fractures a prophylactic cerclage was used, in order to reduce the fragments, before to start with nailing.

**Discussion** Affixus nail is a safe, versatile and very friendly nail. The following items were introduced to improve surgery: the pistol grip to introduce the guide wire, the multihole device to adjust the positioning of the guide at the entry point, the aiming device for lateral targeting of the cervical screw, the retention screwdriver for distal locking screws, the premounted cup screw in the nail, the possibility to use, when needed, an additional antirotational screw in unstable fractures.

**Conclusions** In proximal femur fractures, a correct synthesis must be performed; the cervical screw should be positioned in the centre quadrant in AP and lateral plane. Affixus is a safe nail, and the new instrumentation facilitates surgery. In our hands is the best choice for femoral neck fractures 31A2 and 31A3 type. In 31A1 fractures at the moment a sliding hip screw (DHS) is to be preferred.

### Evaluation report on the process indicator for the treatment of proximal femur fracture in the Turin traumatologic hospital

D. Mellano[1], R. Negretto[1], E. Grosso[1], D. Tangolo[2], M. Panella[3], F. Leigheb[3], K. Vanhaecht[3]

[1]UOSD Chirurgia del Bacino (Turin, IT);
[2]A.O. Città della Salute e della Scienza di Torino (Turin, IT);
[3]European Pathway Association (Belgium)

**Introduction** Hip fractures are a high volume and high socio-economic impact pathology treated in acute care hospitals. Introduction of improvements to the care and its processes, related to this group of patients, may result in a favourable impact on clinical outcome. The purpose of this study is to measure performance of the care process.

**Materials and methods** The EQCP study identified through a systematic review of international literature, key evidence-based interventions for the management of patients with fractures of the proximal femur. In addition, an international panel of experts selected among all the key interventions, through a study of the Delphi methodology, a list of process indicators (including pain assessment, pre-trauma conditions, risk of falls, surgery within 24 h of admission, length of stay, mode of discharge, submission at the centre of osteoporosis treatment, etc.). At the Department of Traumatology CTO Hospital of Turin were included in the study 20 consecutive cases of fracture of the proximal femur. The diagnostic, therapeutic and follow-up of up to 6 months after discharge from the ward was analyzed for each patient.

**Results** Analyzing of the results obtained in performance of process indicators we were able to evaluate the criticality of the entire care process and define possibility of improvement. The results of the new analysis of further measurement after making the necessary corrections, will be available in the forthcoming months.

**Conclusions** The critical analysis of a complicated therapeutic path, as those for the femur fracture, which involves a multi-specialized panel, is complex and it is essential to make the necessary improvements.

### *C05–TRAUMATOLOGY 5*

### Outcome of complex tibial plateau fractures: open reduction internal fixation versus external fixation

V. Conserva, M. Filipponi*, G. Vicenti, V. Russi, B. Moretti

Unità Operativa di Ortopedia e Traumatologia II, Azienda Ospedaliero-Universitaria Policlinico di Bari (Bari, IT)

**Introduction** The treatment of tibial plateau fractures is still controversial in literature, cause these injuries often results in a serious functional impairment with significant implications for the patient. Favourite treatment is almost surgery; stable fixation associated with restore of joint congruity and anatomical axes, preservation of soft tissues are the main issue to consider treating this kind of lesion. Yet open reduction techniques allow better fixation, minimally invasive techniques provide lower damage to the soft tissues.

**Materials and methods** We retrospectively evaluated 42 patients surgically treated for complex tibial plateau fractures (Schatzker IV–VI) at our clinic. Thirteen women, 29 men, mean age 48.1 years, mean follow-up 38.4 months. Thirty patients received open reduction and internal fixation (ORIF) (group 1); 12 patients received closed reduction and external fixation (CREF) (group 2). Thirty were high energy trauma (20 fixed with ORIF; 2 CREF); 12 were low energy trauma (10 fixed with ORIF; 2 CREF). We used Ahlback radiographic score, Rasmussen and WOMAC clinical score and NRS score for residual pain evaluation. Student's $t$ test for independent samples was used for the comparison of means between the two groups. Performance was considered significant for $p < 0.05$.

**Results** Comparison between two groups showed lower time of hospitalization in the group treated with CREF towards ORIF (group 2 7.8 days; group 1 14.2 days); time before surgery was less in the group treated with EF which is correlated with minimal soft tissue damage (group 1 5 days; group 2 3.5 days). WOMAC score resulted better in the group treated with EF (84.2 vs 80.5). Three patients treated with ORIF showed early radiographic sign of arthrosis (Ahlback score). Only four patients showed modest sign of arthrosis. Two patients had soft tissue sign of infection (1 ORIF and 1 EF); three patients developed clear sign of osteomyelitis. Two patients with ORIF had knee contracture at 30° of flexion. We had one screw mobilization in the plate group.

**Discussion** Our data confirm the validity of the various surgical strategies for tibial plate fractures; soft tissue preservation, clinical evaluation and careful choice for the surgery remain the main topic to consider when treating this kind of injury.
**Conclusions** ORIF is involved with excessive soft tissue aggression and a slight delay of functional recovery. Ibrid EF represents a viable option in case of comminuted unstable fractures with soft tissue suffering and vascular nervous disease ensuring lower time of hospitalization, lower risk of infection and lower stiffness especially in elderly patients.

### The UKR knee in fracture outcomes

M. Corbella*, F. Verde, M. Massaro, S. Romagnoli

Istituto Ortopedico Galeazzi (Milan, IT)

**Introduction** Knee prostheses in fracture sequelae are not a routinary surgical procedure. The analysis of the literature shows only seven relevant papers concerning a total of 152 implants, and there's no specific paper related to UNI implants. These data should be read in relation to the poor diffusion of this surgical treatment compared to total knee implants in post-traumatic arthritis.
**Materials and methods** From 1997 to 2007, we evaluated a total of 6,037 implants. We evaluated a total of 236 (3.9 %) cases of post-traumatic sequelae. In case of isolated fractures of the patella with severe degeneration of patello-femoral compartment in patients under 60 years, isolated patello-femoral prosthesis may be indicated. More frequent are cases of partial articular tibial fractures (compartmental). In particular, the fractures of the lateral compartment are more numerous, especially in young patients. In most of the lateral tibial plateau fractures sequelae, there is an indication for a unicompartmental implant.
**Results** The distribution of these cases according to the type of implant shows that the UKR were 57, Bi-UNI 13, standard TKA 81 and semi-hinged prostheses 85. In most cases, therefore, it was necessary to use a standard or semi-constrained prosthesis (70.3 %) while UNI represented 24.1%.
**Discussion** In our series of tibial plateau fracture sequelae involving UNI implants, we had a single failure out of 37 (2.7 %) cases (35 lateral and 2 medial) with a mean follow-up of 6.3 years (2–17 years). The high rate of complications reported in literature in relation to the young age of the patients suggests the difficulty of this treatment.
**Conclusions** Unicompartmental knee implants seem to be an optimal solution in cases of arthritic changes as a result of tibial plateau fracture follow-up in young (<50) patients when conditions permit the treatment.

### Evaluation of the effectiveness of the articular reconstruction after osteosynthesis in fractures of the tibial plateau

N. Ruggieri*, C. Palumbo, G. Pristerà, G. Gargiulo

SCDO Ortopedia e Traumatologia, Presidio Molinette, Cittá della Salute e della Scienza di Torino (Turin, IT)

**Introduction** The best treatment for articular fractures, needing to search the anatomical restoration of the articular surfaces, is internal fixation. However, external fixation is required in open fractures, in closed fractures with severe soft tissue injuries, in highly comminuted fractures, in severe polytrauma as a damage control surgery, in fractures associated with vascular and nervous injury, in unstable dislocations or fractures-dislocations, in fractures of both articular ends, and in unstable, debilitated, anticoagulated, immunosuppressed patients or in patients with malformations or arteriovenous fistulas. This treatment, usually provisional pending internal fixation, when this cannot be done, may be a definitive treatment.
**Materials and methods** The authors present 30 cases (2000–2012) of articular fractures treated with definitive external fixation, aged 17–84 years. The systems used are the Castaman external fixator and the Castaman pelvis fixator by Blue Shark. The cases consist in seven open fractures, three acetabular breakthrough, ten closed fractures associated with vascular injuries, ten fractures in patients with skin disease or other co-morbidities.
**Results** Follow-up was 1–11 years. 29 patients came to bone healing. In one case, septic arthritis required amputation. Ten patients developed post-traumatic arthritis. No one required joint replacement. In one case an arthrodesis was needed.
**Discussion** The aim of achieving a reduction as anatomical as possible is difficult by an external fixation. In order to obtain the best achievable result, a correct assembling design is needed, taking into account: The location of gripping elements based on the fracture and skin conditions. The configuration of fixation assembly foreseeing any second surgical approach made by orthopaedist or by other specialists. The installation must allow: (1) macro- and micro-metric adjustments on gripping elements; (2) secondary addition of other elements; (3) maximum stability complementary surgical gestures can be done such as mini-open, use of pin as levers or K wires as gripping elements for small fragments.
**Conclusions** External fixation can be considered definitive treatment for articular fractures only in special and selected cases. Especially in these cases of particular difficulty, this method has proven to be very reliable and safe, providing primary stability, good anatomical result in the reduction, minimal risk of infection, great mastery in associated soft tissue and vascular nerve injuries, average bone union time of 90 days, and, in addition, this method is essential in limb salvage.

### Angular stable plates in proximal meta-epiphyseal tibial fractures: study of joint restoration and clinical-functional evaluation

S. Giannotti*, M. Scaglione, D. Giovannelli, G. Bugelli, G. Guido

Clinica Ortopedica e Traumatologica II (Pisa, IT)

**Introduction** The angular stable plates are a great solution for treatment of complex closed fractures of proximal tibia. The aim of this study was to evaluate the clinical and functional results obtained through the Knee Society Score and Rasmussen Score in patients treated for this type of fractures with this method. Was also evaluated articular restore through a knee arthroscopy performed in the same time of the removal of the internal fixation devices and subsequently using TC3D reconstruction that have been compared with the preoperative imaging.
**Materials and methods** From January 2009 to December 2012 in our clinic were treated 75 cases of proximal meta-epiphyseal tibial fractures with angular stable plates. In none of the cases of this series have ever made it necessary to perform arthrotomy to achieve reduction of the fracture. The joint recovery has been initiated in the immediate postoperative while the weight bearing with knee brace has been granted not before the 6–8th week. All the patients underwent plate removal (which occurred at an average of 14.66 months from the implant) with arthroscopic and instrumental evaluation with TC3D.
**Results** The clinical and functional evaluation performed by the KSS and Rasmussen Score highlighted the high percentage of good–excellent results (over 90 %). The arthroscopic visualization showed the presence of chondral damage in 100 % of patients analysed with Outerbridge classification. The TC3D subsequently realized at the time of removal of the internal fixation devices have permitted to view the good anatomical reconstruction of the joint and obtain a comparison with the images made at the time of trauma.
**Discussion** The results indicate that angular stable plates you get a good primary stability allowing early joint recovery with an excellent range of motion. We believe that the choice not to perform an arthrotomy of the knee at the time of fracture reduction in this type of injury, could prove to be an advantage in terms of functional recovery. Any surgical treatment of intra-articular lesions associated are delayed at the time of the consolidation of the fracture.
**Conclusions** We believe that the early movement which is achieved by the synthesis provides better functional recovery in addition to a physiological condition that has a positive effect on the evolution of post-traumatic arthrosis.

### Pre-contoured medial plate for distal third tibia fractures

M. Sangiovanni*, I. Felici, R. Buzzi

AOU Careggi (Florence, IT)

**Introduction** Distal metadiaphyseal tibia fractures are notorious difficult to manage. The introduction of locking plate technology has added another treatment possibility. The aim of this study is to evaluate results of 24 distal tibial fractures treated with LCP metaphyseal plate.
**Materials and methods** We retrospectively evaluated 24 distal third tibia fractures (24 patients) including 15 males and 9 females. The mean age was 50.6 years. We used a titanium LCP metaphyseal plate which is anatomically shaped and accepts both locking and non locking screws, 3.5 mm distally and 4.5 mm proximally. Locking screws were used in all cases (at least three distally). Fractures type according to the AO classification was 42 or 43 A in 19 patients (79.2 %), 43 B in 2 patients (8.3 %) and 43 C in 3 patients (12.5 %). There was only a grade II open fracture and in 4 cases (16.6 %) a grade II soft tissue injury. In 15 cases (62.5 %) we employed a short incision technique through a 10 cm incision at the level of the fracture, lateral to the tibial crest. In the remaining 9 patients (37.8 %) the plate was introduced in a subcutaneous tunnel through a short incision over the medial malleolus. Results were evaluated using AOFAS, SF-36, FFI and TEGNER score. Patients were retrospectively followed for average duration of 43.87 months (range 14–97).
**Results** There were no early complications (infection, wound dehiscence, DVT). According to the AOFAS, scores were 13 excellent (54.2 %), 10 good (41.7 %) and fair in only 1 patient (4.2 %) with a moderate reduction in flexion–extension and eversion–inversion. The average FFI score was 2.79 % (range 0–100 %); the average Tegner score before injury was 5.4 and 5 at follow-up; the average SF-36 score was 80.75 (range 0–100). Reduction on the post operative radiograph was anatomic in 13 patients (54.2 %) and near anatomic in 11 patients (45.8 %). There were no cases of loss of fixation, nonunion or osteomyelitis. In two cases plate was removed.
**Discussion** The results using locking plate in the distal tibia were positive with a low incidence of complications. The locking screws are useful to stabilize short distal fragments; their use with a MIPO technique was without primary loss of reduction and minimize soft tissue damage and devascularization of the fractures fragments, respecting bone's biology and providing biomechanically stable construct.
**Conclusions** This study demonstrates that excellent results can be achieved with a locking plate in this difficult fractures.

### Dynamic locking screws in treatment of closed distal metaphyseal tibia fractures: a multicenter prospective study

G. Vicenti*[1], V. Conserva[1], C. Mori[1], N. Tartaglia[2], L. Moretti[1], V. Pesce[1], B. Moretti[1]

[1]Unità Operativa di Ortopedia e Traumatologia II, Azienda Ospedaliero Universitaria Policlinico di Bari (Bari, IT);
[2]Unità Operativa di Ortopedia e Traumatologia Miulli (Acquaviva, IT)

**Introduction** Minimally invasive technique (MIPO) seems more advantageous for soft tissues and bone biology, prolonged healing was observed in simple fracture pattern when a bridging plate technique was used. Fracture consolidation with LCP requires interfragmentary movement to stimulate callus formation. The high stiffness of locked constructs is recognized as a cause of deficient healing; the near cortex is particularly predisposed to deficient healing. Dynamic locking screws (DLS) reduce primarily the axial stiffness of LCP osteosynthesis, while the bending stiffness is less manipulated; interfragmentary movement increase on the near cortical side.
**Materials and methods** We randomized 20 patients affected by distal tibia fracture, treated with mini-invasive bridging plate technique and DLS on the proximal side of the fracture (group 1). We monitored time of consolidation, quality of the reduction, complications and AOFAS score by comparing the results with those from a control group treated with homogeneous LCP and standard screws only (group 0). Student's $t$ test for independent samples was used for the comparison of means between the two groups. A multiple logistic regression model was constructed to assess the possible confounding effects. Performance was considered significant for $p < 0.05$.
**Results** The study sample consists of 40 subjects, 20 per group. Minimal follow up was of 6 months, maximum of 24. AOFAS score was 89.65 for group 1 (DLS) and 85.50 for group 0 (LS). The average healing time is of $17.6 \pm 2.8$ weeks in the group treated with standard screws and $13.5 \pm 1.8$ weeks in the group treated with DLS ($t = 5.5$, $p < 0.0001$). Multiple logistic regression model showed the linkage between healing time and association with group 1 ($t = 5.31$, $p < 0.0001$). Comparison between simple fracture pattern (42-A; 43-A) of both group showed a faster healing process in DLS construct (group 1) supported by statistical significativity.
**Discussion** LCP with MIPO technique and bridging configuration, represents a viable method for the treatment of simple fractures distal third of the tibia. Yet literature describes a 20 % of delayed union and nonunion. The construct is often too stiff especially on the near cortex where micromotion is not enough to promote the necessary trophic stimuli for the healing process. Our results accord the literature in patients receiving standard LCP fixation.

**Conclusions** DLS with mininvasive plate osteosynthesis in a bridging construct for simple meta-diaphyseal bone fractures allows modulation of the rigidity of the system and facilitates development of early and triplanar callus.

### *C06–TRAUMATOLOGY 6*

### Advantages of internal synthesis with plate in the treatment of calcaneus fractures: a retrospective study and our experience

P. Mantelli*, S. Cervi, L. Bisogno

Presidio Ospedaliero Val d'Arda, Azienda USL Piacenza (Fiorenzuola d'Arda, IT)

**Introduction** The different types of approach, as well as not unique indications, make the treatment of calcaneus fractures a strongly current topic. The purpose of this paper is to describe the advantages of internal plate synthesis, in our experience, compared to other methods in the displaced calcaneus fractures.
**Materials and methods** From January 2010 to December 2012, we have treated surgically 38 patients, come to our observation with an intra-articular complex calcaneus fracture. We retrospectively selected two homogenous groups of ten patients by sex, medical conditions before trauma and type of fracture. The first group was treated with percutaneous Kirschner wires, the second group with open-reduction and internal fixation with plate and screws. Only cases in which we achieved a satisfaction postoperative X-ray result were included in the two selected groups. We evaluated VAS scale, post-operative and follow up Bohler's angle, post-operative complications and a podoscopic evaluation was made.
**Results** We found a radiographic stability in the group treated with plate, corresponding to the decrease in the VAS scale during walking mostly after the third month after surgery; patients undergoing percutaneous synthesis with Kirschner wires, in the same period, showed a progressive decrease of Bohler's angle, associated with a pain increasement.
**Discussion** About clinical and radiographic evaluation, we believe that in calcaneus fractures the plate osteosynthes is better than less invasive system like K-wires, especially in long term follows-up because it prevents the thalamus collapse and prevent functional complications like flat feet.
**Conclusions** The open reduction and internal calcaneus fixation is a complex surgical procedure and not without complications but it can guarantee a better correction of deformity and a good fracture stability.

### Intra-articular calcaneal fractures: surgical treatment vs. conservative treatment

D. Leonetti*[1], L. Amendola[1], P. Barca[1], S. Comitini[2], M.T. Miscione[3], F. Gonella[1], L. Prosperi[1], D. Tigani[1]

[1]Ospedale Maggiore Bologna (Bologna, IT);
[2]Università degli Studi di Catania, Ospedale V. Emanuele (Catania, IT);
[3]Istituto Ortopedico Rizzoli, Università di Bologna (Bologna, IT)

**Introduction** Calcaneal fractures account for about 60 % of tarsal fractures and 2 % of all fractures. In 80–90 % of cases, these lesions affect young and active people, and represent a high cost in terms of socio-economic development because approximately only 20 % of patients is able to return to work before 12 months. Due to the poor results and to several complications, treatment of these lesions is still much debated. The aim of this study is to evaluate retrospectively a series of 69 cases of intra-articular calcaneal fractures by comparing the outcomes obtained by surgical treatment compared to the results obtained by the conservative treatment.
**Materials and methods** From 2009 to 2011, 69 patients, affected by Sanders type II–III intra-articular calcaneal fractures, were treated in our hospital. Forty-two cases were treated surgically and 27 cases conservatively. All patients were evaluated clinically and radiographically by CT and Rx at the time of trauma. They were then evaluated monthly for the first 3 months and then at 6, 12 months and date of last follow-up. The clinical evaluation was performed by the use of the AOFAS score indicating the values between 90 and 100 as excellent results, between 75 and 89 as good, between 50 and 74 as fair and less than 50 as poor.
**Results** All fractures healed within 6 months from the date of the trauma. The clinical results were good or excellent in more than 75 % of surgically treated cases and in 30 % of cases treated conservatively. More than 90 % of surgically treated patients have returned to work before 12 months. In two cases treated by surgical reduction and internal fixation with plate there was a dehiscence of the surgical wound.
**Discussion** The treatment of intra-articular calcaneal fractures represents a challenge for the orthopaedic surgeon. The main goal of surgical treatment is to reconstruct anatomically the subtalar joint surface and the calcaneal morphology. The surgical treatment that fails this objective and the conservative treatment often lead to unsatisfactory results.
**Conclusions** Surgical treatment in Sanders type II–III intra-articular calcaneal fractures it allows an anatomic or almost anatomical reduction of the fracture and good clinical results in more than 75 % and also allows an earlier return to work than to conservative treatment.

### LCP distal lateral plate in the treatment of fibular fractures: our experience

L. Turelli[1], A. Mondaini*[1], L. Nistri[2]

[1]Ospedale S. Maria Annunziata, ASL 10 Firenze (Florence, IT);
[2]C.T.O. Università degli Studi di Firenze (Florence, IT)

**Introduction** Fibular fractures are very frequent in traumatology. Sometimes they are associated to tibial malleolus fracture, seldom are isolated. In case of displacement they need surgery. The anatomical reduction is very important because the displacement (even with the deltoid ligament integrity) may cause tibio-talar incongruence and an early arthritis. ORIF is today the golden standard.
**Materials and methods** From January 1st 2011 to December 31st 2012, 67 distal fibular fractures using LCP distal lateral plate were treated. We evaluate clinical and radiological results after 6 months from the surgery.
**Results** In one patient the plate had to be removed because of an infection after 2 months. 66 cases healed with total weight bearing after 3 months.
**Discussion** We studied the advantages of this kind of plate compared to 1/3 tubular plate and traditional LCP one. The first one is very easy to use thanks to its formability but it doesn't offer a good stability, overall in case of distal fractures. In fact, in this case, sometime we can use only two distal screws (one of these is often a cancellous screw) with minimal stability. On the other hand the LCP offers a good strength sensation due to the angular stable locking system, but in case of soft tissue damages or in case of skin deficiency (for example post traumatic oedema) appears to be sometimes thick and the skin incision healing may not occur.

**Conclusions** We partly solved these problems using the LCP distal lateral fibula plate. The distal part of the plate permits to fix the fragments with more than two angular stable screws increasing the stability. On the other side this plate is less thick than LCP one with decreasing of healing skin complications.

### Talar fractures: treatment with open reduction and internal fixation

S. Bianco*, F. Tonelli, L. Bettini, R. Buzzi

AOUC (Florence, IT)

**Introduction** Talar fractures are uncommon lesions representing less than 1 % of all fractures. Usually are consequence of motor vehicle accidents or falls from height. Complications are frequent and are critical to tibio-tarsal, subtalar and midtarsal joints biomechanics. The aim of this study is to evaluate clinical results and complications of surgery treatment in body, neck and head talar fractures.
**Materials and methods** Thirty-four patients with 35 central talar fractures treated with open reduction and stable fixation from 1996 to 2011 were retrospectively evaluated. Thirty-two patients (33 fractures) were examined at follow-up. Clinical results were evaluated by the American Orthopaedic Foot and Ankle Society questionnaire. Post-traumatic arthritis and osteonecrosis of talar body were assessed with foot and ankle weight-bearing X-ray.
**Results** Six cases (21 %) with radiographic signs of osteonecrosis in 29 body and neck talar fractures were observed. In two cases we observed a partial collapse of the talar body. The average time between trauma and surgery was 6.7 days. We observed no correlation between surgical delay and development of osteonecrosis. Radiographic signs of tibiotarsal, subtalar and talo-navicular arthritis were observed in 27 of 33 fractures (81 %). The sub-talar joint was the most involved joint (68 %). Higher incidence of post-traumatic arthritis was observed in talar body fractures than in talar neck fractures. Talar body arthritis was more severe. The average clinical results were good. Poor results were a consequence of pain and functional limitations.
**Discussion** Talar fractures are complex lesions frequently leading to poor outcomes. Osteonecrosis and post-traumatic arthritis are the most common complications. In this study post-traumatic arthritis of the subtalar joint was the most frequent complication; no relation were noticed between osteonecrosis and surgical timing. Pain and functional limitation of the three joints influenced negatively clinical results.
**Conclusions** Surgical treatment is mandatory in body, neck and head talar fractures because these lesions need anatomic reduction and stable fixation. Despite careful reduction and stable fixation satisfactory result cannot be obtained in all cases, so patients must be adequately informed about possible complications and poor outcome.

### *C07–TRAUMATOLOGY 7*

### Locked plate and mini-invasive technique in proximal humerus fractures: clinical results of a prospective study

S.A. Ghoreishi*, G. Manetti, A. Raspanti, F. Tonelli

AOU Careggi, CTO (Florence, IT)

**Introduction** Many surgical approaches and implant systems are available for the treatment of proximal humerus fractures. This prospective study was carried out using the NCB-PH system (non-contact-bridging for proximal humerus, Zimmer) with a minimally invasive approach (deltoidal split anterolateral to acromion).
**Materials and methods** From November 2009 to January 2013 31 patients (32 fractures) affected by proximal humerus fracture were operated by a single senior surgeon with the NCB-PH plate through a minimally invasive approach. Clinical and radiological outcomes were evaluated with Constant and DASH scores.
**Results** Average age of patients was 62.8 years (range 34–86 years). Fractures were classified according to AO and Neer classifications. Average follow-up was 8.7 months (range 2–25 months). Average Constant score was 74.6 points (range 43–100); average DASH score was 25.3 points (range 0–85). No cases of permanent or transitory axillary nerve lesion were reported. No cases of superficial or deep infection as well as cases of non-union or pseudoarthrosis were experienced in our study. We reported one case of avascular necrosis of the humeral head with consequent secondary screw perforation.
**Discussion** Clinical evidence in literature points out the validity of minimally invasive anterolateral to acromion approach. Our results, using NCB-PH plates through this kind of approach, reflect the actual general trend. Angular stability permits an adequate synthesis in the majority of patients with osteoporotic bone therefore allowing early intensive rehabilitation protocols in the post-operative period. Moreover it is related to a lower incidence of loss of reduction and non-union. This technique indeed allows to obtain good reduction of fragments and minor stripping from their soft tissue attachments therefore decreasing the risk of avascular necrosis of humeral head (which is increased by factors as age of patients, kind of fracture, trauma-surgery time etc.). This technique requires a large learning curve however. Moreover surgeons must carefully avoid axillary nerve lesions.
**Conclusions** In our experience the use of NCB-PH plates through mini-invasive trans-deltoidal approach provides good results in the treatment of proximal third humeral fractures. This technique can be also used in complex fractures with good clinical-functional results.

### Treatment with O'Nil plate in 3–4 part fractures of the proximal humeral epiphysis

E. Bonicoli*, G. Digrandi, I. Castellini, L. Andreani, N. Piolanti, M. Lisanti

Ortopedia e Traumatologia I Universitaria (Pisa, IT)

**Introduction** Proximal humeral fractures are the 5–9 % of all the fractures and 20 % of these need surgical treatment. There are different surgical strategies ranging from percutaneous fixation, intramedullary nail, ORIF to prosthetic replacement. The choice depends on vary factors, such as the pattern of the fracture, the age of the patient and the surgeon experience.
**Materials and methods** From September 2009 to December 2012 in the I Orthopaedic Department of Pisa University were treated with plate fixation 35 proximal humeral fractures in 34 patients. Sixteen were 3-part fractures while 14 were 4-part ones. Mean age of the patients was 59 years (range 29–87), 24 were female and 12 were male. Surgical approach was the deltopectoral in all cases. From the first post-operatory day shoulder mobilization was allowed with pendular movement and abduction till 50°, compatibly with pain. For 2 weeks patients wore a shoulder brace with 15° of abduction. After 3 weeks active shoulder mobilization was allowed. Mean follow up was 13.2 months (range 1–41). Patients was clinically and radiologically followed; also functional scores were performed (Constant shoulder score, UCLA shoulder rating scale and DASH score). The implant was the O'Nil proximal humerus fixator, an angular stability plate made by Intrauma.

**Results** During the follow-up we do not report early or late infections, neurovascular lesions or devices ruptures. We report two bad results: a case of avascular necrosis of the humeral head, happened after 10 months, treated with prosthetic replacement and a case of early mobilization of two proximal screws that required a new surgical treatment.
**Discussion** Plate osteosynthesis of proximal humeral fractures s requires right indications and selected patients. We think that a good fracture reduction and a stabile implant fixation are indispensable standards in order to start early physiotherapic treatment. On the contrary, long shoulder immobilization, may lead to a limitation of the functionality also in case of good fracture reduction.
**Conclusions** Plate osteosynthesis in proximal humeral fractures is a valid surgical solution. The kind of implant is not yet codified and it is still a debated issue. Our experience with this type of angular stability plate in 3–4 part fractures is certain positive.

### Elbow fractures-dislocation treatment with articulated external fixation: preliminary and clinical-functional results

D. Chiapale*, S. Briano, G. Demontis, S. De Rosa, M. Stella, F. Visci, F. Santolini

Genoa, IT

**Introduction** Fracture-dislocation of elbow involving the radial head and coronoid process, are fractures historically difficult to treat and manage in view of the bad functional results.
**Materials and methods** Authors report their experience in the treatment of fracture-dislocation of the elbow with external fixator (FE) STAR 90 F4 Motion, placing their attention on the surgical technique and its particular indication.
**Results** FE is able to restore the temporary stability of the elbow in a dynamic manner ensuring the healing of capsulo-ligamentous structures.
**Discussion** Preliminary data show that the articulated elbow STAR 90 FE F4 Motion, through a concentric stability permits healing in a dynamic way without causing frequent outcomes of instability, stiffness and pain, and this data is also supported by the X-ray study that shows the presence of fibro-calcific stripes in correspondence of damaged ligamentous structures sign of a independent biological reconstruction.
**Conclusions** The FE articulated elbow enjoy all the advantages of a static setting with the benefits of a continuous movement and in most plans (flexion–extension and pronation–supination), as opposed to other techniques, it has allowed an early joint recovery, an improvement in pain symptoms and an early return to work.

### *C08–TRAUMATOLOGY 8*

### Fractures of the glena fixation with a mixed open-arthroscopic technique

D. Pili*, A. McKee, A. White, D. Deriu

Peterborough and Stamford Hospitals (Peterborough, UK)

**Introduction** The treatment of the intraarticular fractures of the glena is quite challenging due to the difficulties visualising the fracture when trying to achieve the reduction and for the technical difficulties during the fixation with the only aid of fluoroscopy. These difficulties makes these kind of surgery challenging and the results are often poor. The intent of this article is to describe a novel approach to these fractures with the use of a combined technique open-arthroscopic and with the aid of arthroscopic pin guides. This approach to these fractures makes the operation more comfortable for the surgeon and safer so that the aimed result of the procedure is easier to achieve.
**Materials and methods** Though a posterior portal an arthroscope is introduced into the shoulder joint and the fracture reduced under vision. Also the placement of cannulated screws is performed under arthroscopic vision with the aid of variable angle guides.
**Results** With this method the reduction of the articular fracture can be thoroughly assessed intra-operatively and clearly visualised. The introduction of the metalwork for the internal fixation is also quite simple and safe.
**Discussion** The intra-articular glena fractures are difficult to treat and significant loss of function can follow such injuries. To avoid the loos of function end the development of post-traumatic complications the reduction of the fracture and the fixation should be as accurate as possible. These results can be achieved with this mixed technique open/arthroscopic.
**Conclusions** The technique described in this article demonstrated a successful fracture reduction and a stable fixation. This technique is easily and safely executed by the shoulder arthroscopy surgeon and therefore easily employed.

### Distal radius fractures treated with DVRA plate: clinical and radiographical outcome

M. Del Torto*, R. Politano, A. Maresca, R. Pascarella

Ortopedia e Traumatologia, Ospedali Riuniti (Ancona, IT)

**Introduction** Articular distal radius fracture is the most common trauma of the upper limb. In recent years we assisted to a progressive extension of surgical indications of wrist fractures. Surgical treatment of wrist fractures aims to reach a double objective: anatomical reduction and stable synthesis. A dorsal approach for plating reports frequent complications, such as loss of reduction and extensor tendon lesions, while volar approach reports less complications. Volar plating with locking screws showed best results in terms of stiffness and stability in comparison to standard plates.
**Materials and methods** Aim of our study is to evaluate clinical and radiographical results of patients treated with DVRA volar plate. We treated 121 patients affected by wrist fracture. Fifty-two female and 69 male. Average age was 52 years old (range 25–82). Thirteen fractures in patients with polytrauma e 18 patients poyfractured. Ninety-five cases were articular fractures. Average follow up was of 20 months. A radiographical and clinical evaluation through Gartland–Werley Score was performed.
**Results** The score obtained was excellent in 51 cases, good in 39 cases, fairly good in 19 cases and poor in 12 cases. Radiographical results were good in terms of volar tilt, radial angle and ulnar variance. Seven cases of failure: two caused by subchondral bone failure, two caused by screw mobilization and three caused by screws in articulation.
**Discussion** This plate shows a good adaptability to volar radius surface with the possibility of radial styloid and dorsal fragments synthesis through angular stability. Plate profile surrounds watershed line respecting principles of flexor tendon not irritation and volar support. Screws positioning range allows to reach subchondral bone of distal radius.
**Conclusions** The possibility of a early mobilization and the possibility of an only surgical way are characteristics that make this plate a good alternative for orthopaedic surgeons.

### Surgical repair of distal biceps brachii tendon rupture

E. Siuni*[1], G. Sulcis[2], V. Pace[1], A. Mastellone[1], I. Pusceddu[1], D. Nonne[2]

[1]Servizio Chirurgia della Spalla, SCOMR, ASL Cagliari (Cagliari, IT); [2]Scuola di Specializzazione Ortopedia (Cagliari, IT)

**Introduction** The present study consisted in a clinical follow-up of patients with distal rupture of the biceps brachii tendon managed by suture anchor reinsertion to the radial tuberosity.

**Materials and methods** From 2010 to 2012 we treated 12 patients with traumatic distal biceps brachii tendon rupture. All patients were men; mean age was 38 years: 7 was heavy working and 5 was bodybuilder. We used the single anterior incision technique: the tendon readaptation to its origin was done by a suture metal anchor. Post-operative immobilization was for 3 weeks in a cast or splint at 90°–110° of elbow flexion and neutral prono-supination, then protected passive and active motion for additional 3 weeks and physiotherapy 2 months at least.

**Results** The results was evaluated by clinical, radiological and ultrasound exams and SECEC Elbow Score. Nine patients have regained the complete range of flexion–extension of the elbow; we found one patient with flexion deficit (10°) and two patients with extension deficit (5°) compared with against side. All patients have recovered their full pronation; two of all reported a supination deficit (5° and 10°). Patient satisfaction and overall muscle strength recovery were very good. Two patients reported discomfort during heavy manual jobs. Return to ADLs was at a mean 3 months and all patients returned to previous work or sport at a mean 4 months. We found two cases of reversible lateral antebrachial cutaneous nerve paresthesia.

**Discussion** Distal biceps tendon rupture is an injury typically reported in the dominant extremity of middle-aged men who relates a history of lifting an object or a forced extension with a flexed and loaded forearm. Conservative treatment causes a decrease of flexion strength of the elbow, decrease of supination strength of the forearm and loss of normal anatomic contour of the biceps. Surgical treatment allows a greater recovery of the function of the biceps brachii.

**Conclusions** The surgical treatment and anatomic repair is an efficient option in the distal biceps tendon rupture, especially for patients with high functional demands, workers or sportsman. The technique with single anterior incision and fixation with two suture anchors provides good functional outcomes with good satisfaction for the patients.

### Repair of acute distal biceps tendon ruptures using the Arthrex system

M. Federici*, F. Isoni, F. Lanza

Ospedale Santa Corona, ASL 2 Savonese (Pietra Ligure, IT)

**Introduction** The rupture of the distal tendon of the biceps brachii is an uncommon lesion and accounts for about 10 % of all the injuries of the biceps. The literature on this topic remains scarce and the best surgical treatment controversial. There are several methods of reconstruction and fixation of the tendon. The aim of this study was to evaluate the results obtained by the authors in a consecutive series of patients treated with Distal Biceps Repair System (Arthrex).

**Materials and methods** We conducted a retrospective study of 14 patients of mean age 54.6 years (range 47–63) operated between June 2010 and December 2012. The surgery was performed by the same surgeons with a single-incision technique and fixation obtained using the system Distal Biceps Repair (Arthrex). The post-operative protocol included rest with arm in a sling for 15 days, gradual recovery of elbow flexion and extension; the activities of everyday life were allowed after 1 month and the sports activity was allowed at 6 months. For clinical evaluation we used the evaluation board DASH (Disabilities of the Arm, Shoulder and Hand).

**Results** One patient was lost at follow up. Thirteen patients were evaluated with an average follow up of 9.4 months (range 6–24) and the mean DASH score recorded was of 12.3 (range 4.2–25.3). Twelve patients returned to their previous level of activity; in one case a second surgical procedure was necessary to remove the FiberWire suture for septic complication; in two cases we found heterotopic ossification. In one case we had a temporary posterior interosseous nerve palsy, recovered in 6 months.

**Discussion** Among the different procedures available the technique used by the authors has demonstrated its validity due to the simplicity of the surgical act; the intimate adhesion of the tendon to the bone allows healing; furthermore stable fixation can ensure early mobilization and then good outcomes.

**Conclusions** In the light of the results obtained, the surgical treatment of distal biceps tendon in patients with high functional demands is a valid therapeutic option. The technique used is proved to be simple, safe and effective.
